# Survival, Treatment Outcome, and Safety of Multiple and Repeated Courses of Stereotactic Body Radiotherapy for Pulmonary Oligometastases of Head and Neck Squamous Cell Carcinoma

**DOI:** 10.3390/cancers15215253

**Published:** 2023-11-01

**Authors:** Samuel Moritz Vorbach, Julian Mangesius, Daniel Dejaco, Thomas Seppi, Matthias Santer, Stephanie Zur Nedden, Manuel Paolo Sarcletti, Martin Josef Pointner, Tilmann Jakob Hart, Herbert Riechelmann, Ute Ganswindt, Meinhard Nevinny-Stickel

**Affiliations:** 1 Department of Radiation-Oncology, Medical University of Innsbruck, 6020 Innsbruck, Austria; samuel.vorbach@i-med.ac.at (S.M.V.); thomas.seppi@i-med.ac.at (T.S.); manuel.sarcletti@i-med.ac.at (M.P.S.); martin.pointner@i-med.ac.at (M.J.P.); tilmann.hart@i-med.ac.at (T.J.H.); ute.ganswindt@i-med.ac.at (U.G.); meinhard.nevinny@i-med.ac.at (M.N.-S.); 2 Department of Otorhinolaryngology—Head and Neck Surgery, Medical University of Innsbruck, 6020 Innsbruck, Austria; daniel.dejaco@i-med.ac.at (D.D.); matthias.santer@i-med.ac.at (M.S.); herbert.riechelmann@ikbnet.at (H.R.); 3 CCB-Biocenter, Institute of Neurobiochemistry, Medial University of Innsbruck, 6020 Innsbruck, Austria; stephanie.zur-nedden@i-med.ac.at

**Keywords:** pulmonary oligometastases, head and neck squamous cell carcinoma, stereotactic body radiotherapy, repeated courses of SBRT

## Abstract

**Simple Summary:**

The objective of this retrospective study was to evaluate whether stereotactic body radiotherapy (SBRT) is safe and effective when applied for the treatment of oligometastatic pulmonary metastases (PM) exclusively derived from head and neck squamous cell carcinoma (HNSCC). Excellent local control of 92 treated single and multiple lesions was achieved even after repeated courses of SBRT of newly emerging metastases. Overall survival of the 46 included patients was in the upper range of previously reported results. Toxicities were limited to grade 2 and no functional lung impairment was observed. Based on the presented findings in this largest investigated cohort of exclusively HNSCC-derived PMs to date, SBRT can be recommended as a safe and effective treatment alternative to surgical resection for inoperable and resectable lesions.

**Abstract:**

Current literature regarding survival and treatment outcome of SBRT in patients with pulmonary oligometastatic head and neck squamous cell carcinoma (HNSCC) is limited. Additionally, most of the published studies include metastatic lesions deriving also from primaries with histologies other than SCC when investigating the outcome of SBRT. The aim of the present retrospective study is to explore local control (LC) of treated metastases, progression-free survival (PFS), and overall survival (OS) of exclusively pulmonary oligometastatic HNSCC-patients treated with SBRT. Between 2006 and 2021, a total of 46 patients were treated with SBRT for a maximum of four pulmonary oligometastases (PM) concurrently (mean PM per patient = 2.0; range 1 to 6 PM, total of 92). Of these, 17 patients (37.0%) developed new pulmonary metastases after their first SBRT. Repeated courses of SBRT were required once in 15 patients (88.2%) and twice in 2 patients (11.8%). Median follow-up was 17 months (range, 0–109 months). One year after completion of SBRT, LC rate, PFS, and OS were 98.7%, 37.9%, and 79.5%, respectively. After two years, LC rate, PFS, and OS were 98.7%, 28.7%, and 54.9%; as well as 98.7%, 16.7%, and 31.0% after five years. Radiochemotherapy (HR 2.72, *p* < 0.001) or radiotherapy as primary treatment (HR 8.60; *p* = 0.003), as well as reduced patient performance status (HR 48.30, *p* = 0.002), were associated with lower PFS. Inferior OS correlated with poor performance status (HR 198.51, *p* < 0.001) and surgery followed by radiochemotherapy (HR 4.18, *p* = 0.032) as primary treatment, as well as radiotherapy alone (HR 7.11, *p* = 0.020). Treatment of more than one PM is an independent predictor of impaired OS (HR 3.30, *p* = 0.016). SBRT of HNSCC-derived PMs results in excellent LC rates and encouraging OS rates of 54.9% at two years along with good tolerability (no more than grade 2 toxicities). Favourable outcome and low toxicity also apply to repeated courses of SBRT of newly emerging PMs.

## 1. Introduction

Head and neck squamous cell carcinoma (HNSCC) ranks as the seventh most common tumour globally, leading to around 800.000 new cases each year [1], and the overall incidence continues to rise [2]. Currently available curative treatment options include radiation therapy with or without chemotherapy or surgical resection with or without postoperative irradiation. While for localized HNSCC, unimodal treatment, consisting of either radiation therapy without chemotherapy or surgical resection without postoperative irradiation, is administered, locally advanced or advanced HNSCC requires multimodal treatment, combining either radiation therapy with chemotherapy or surgical resection with postoperative irradiation [3,4,5].

With the development of more effective treatment options, the long-term loco-regional control rate has reportedly increased up to 75% [6]. Especially, volumetric modulated arc therapy (VMAT), a radiation technique that can achieve highly conformal dose distributions, effectively covers the target volume while sparing the normal tissue [7], thus leading to increasing control of the primary tumour. In addition, improved staging and the relative increase in HPV-driven oropharyngeal cancer [8], which is characterised by high radio- and chemosensitivity [9], demonstrated to be beneficial for HNSCC patients. Before that, later appearing distant metastases were a rare occurrence due to the high frequency of early fatal loco-regional failure. Approximately 3% of patients present with distant metastases at initial diagnosis [10], but 20–30% of patients currently develop distant metastases later in the course of their disease [11,12,13,14]. To summarize, the primary obstacle to further improve OS beyond the current 50% margin at five years [15] is increasingly shifting from failure in locoregional control to metastatic disease.

The concept of oligometastatic disease, originally defined by Hellman and Weichselbaum in 1995, is an intermediate state between localized and advanced disease [16]. Currently, oligometastatic disease is mainly described as having ≤5 accessible metastatic lesions that can potentially be managed using localized strategies [17]. Among local ablative treatments, stereotactic body radiotherapy (SBRT, also known as stereotactic ablative body radiotherapy, SABR) is an increasingly used non-invasive treatment option with local control rates of over 90% for lesions in the lung, accompanied by good tolerability and safety, with most studies reporting grade 3 toxicities in around 5% of the patients [18,19].

The lung is the most common site of metastasis in HNSCC, accounting for 60–70% of the distant metastases [20,21]. Although ablative local treatment modalities are recognized as feasible curative treatment option for PMs in oligometastatic HNSCC by the National Comprehensive Cancer Network and the European Society for Medical Oncology, currently available data about SBRT’s prognostic implications are limited and frequently underpowered. In addition, the majority of these studies also included various histologies other than HNSCC as well as other organs affected by metastases [22,23,24,25,26,27,28,29].

The aim of this study was to provide a comparatively large data set evaluating the promising therapeutic benefits of multiple and partially repeated courses of SBRT for the treatment of exclusively HNSCC-derived pulmonary metastases (*n* = 92). In addition, we are the first to present outcome, local control rates, and toxicity data from a subset of patients who received SBRT more than once to treat successfully also repeatedly developed new pulmonary metastases during the course of their disease.

## 2. Materials and Methods

### 2.1. Study Population

Patients with pulmonary metastases who were treated with SBRT at the Department of Radiation Oncology, Medical University of Innsbruck, Austria, between August 2006 and February 2021, were retrospectively identified. Patients with histologically verified pharyngeal, laryngeal, or oral HNSCC were selected from this group.

Demographic factors were recorded at initial diagnosis and at the time of SBRT and included age in years, sex, and smoking history. The Eastern Co-operative Oncology Group (ECOG) score was used to assess patients’ functional status and ability to self-care [30]. The medical data recorded included the site of the tumour, the AJCC TNM classification valid at the time of diagnosis, the primary treatment, and the time from the initial diagnosis to the diagnosis of pulmonary metastases. To obtain p16 status for patients with oropharyngeal cancer, surgical specimens were prepared as previously published [31]. The cut-off for p16 positivity was ≥70% positive tumour cells.

All cases were discussed in the institutional multidisciplinary tumour board. All patients received curative-intent standard-of-care treatment of their primary HNSCC (radiation therapy with or without chemotherapy or surgical resection with or without postoperative irradiation), as recommended by the institutional multidisciplinary tumour board. Following the development of lung metastases, CT-guided puncture was routinely performed for histological verification of lung lesions. Spirometry was performed to ensure patient eligibility for CT-guided puncture. Patients were re-submitted to the tumour board with the histological report and assigned to SBRT. All patients in our cohort had a controlled primary tumour at the time of SBRT, except for two patients, who were newly diagnosed with pulmonary metastasis already during the therapy of their primary tumour. For all patients, pulmonary metastases were the only diagnosed metastatic lesions at the time of SBRT and they were all treated radically with curative intent.

The study has been approved by the institutional review board of the Medical University of Innsbruck (EC no. approval: 1384/2022). All procedures conducted in this study involving human participants were in accordance with the ethical standards of the institutional review board as well as with the Helsinki declaration (1964) and its later amendments or equivalent ethical standards.

### 2.2. Techniques of Radiotherapy

Elekta BodyFIX was used to immobilise patients in the supine position. A four-dimensional CT scan was acquired to capture the location and movement of the tumour over time. An internal target volume (ITV) was defined to account for the effects of respiratory motion on the clinical target volume (CTV). In the case of multiple metastases, a cumulative CTV (CTV_total_) was calculated and used for comparative analyses. The creation of a planning target volume (PTV) involved uniformly expanding the ITV. Organs at risk (such as the lungs, spinal cord, trachea, bronchial tree, oesophagus, chest wall, and blood vessels) were outlined to minimise radiation exposure to the greatest extent achievable. Depending on the proximity of the lesion to the organs at risk, different dose concepts were applied: 60 Gy in 10 fractions (13.6%), 48 Gy in 6 fractions (19.4%), or 45 Gy in 3 fractions (67%). The according dose prescription modalities included the 65% isodose, the 80% isodose, or the 100% isodose. Treatment planning was performed using precisePLAN (Elekta AB, Stockholm Sweden) until 2013, and Pinnacle Software (most recent version v14; Philips Medical, Fitchburg, MA, USA) until the end of the study. Patients were treated using three-dimensional conformal radiation therapy (3D-CRT) or VMAT using an Elekta Synergy linear accelerator until 2013, and a Versa HD linear accelerator since then (both from Elekta AB, Stockholm, Sweden). Daily cone beam CT scans were used to assess and, if necessary, to correct patient position to ensure PTV inclusion of metastases before each session.

### 2.3. Follow-Up

After completion of SBRT, follow-up radiological imaging (usually CT scan, if necessary PET-CT) was performed every 3 months for 1.5 years and then every 6 months thereafter. The interdisciplinary tumour board evaluated any unclear finding and, if necessary, a shorter-term control including a CT scan was initiated. Tumour response was classified by two independent radiologists with more than 15 years of experience, according to RECIST (response evaluation criteria in solid tumours) [32]. Follow-up time was defined as the time between the SBRT and the final CT scan. Local control was defined as progression of the treated lesion and measured as the time from the end of SBRT to progression or to the final follow-up. PFS was defined as the time from the end of SBRT to progression in the treatment field or outside. Overall survival was defined as the time from the end of SBRT to either death from any cause or the date of the final follow-up. Toxicity was monitored by anamnesis, medical examination, laboratory test, and medical imaging and classified according to the common terminology criteria for adverse events (CTCAE version 3.0-5.0 [33]).

### 2.4. Statistical Analysis

The endpoints of this study were local control rate of treated metastases, progression-free survival, and overall survival. Statistical analysis was conducted using SPSS Statistics (V26, IBM Corporation, Armonk, NY, USA). Descriptive analysis was used to provide summaries of relevant patient and treatment characteristics. Spearman’s rank correlation coefficient was used to test for correlation between the observed toxicities and the CTV size or the mean lung dose. LC, OS, and PFS were calculated using the Kaplan–Meier method. The Cox proportional hazards model was used for the univariate and multivariate analysis of the factors and their hazard ratios with 95% confidence intervals associated with PFS and OS. Multivariate analysis was performed by applying the rule of stepwise backward eliminations of non-significant factors. The *p* values < 0.05 were considered to be significant. The linear quadratic model was used to calculate respective biologically effective doses (BEDs) for all radiotherapy prescription doses with an assumed alpha/beta ratio of 10 (BED10).

## 3. Results

### 3.1. Patient Population

This study included 46 patients with a total of 92 pulmonary lesions treated with SBRT from August 2006 to February 2021. The median follow-up time was 17 months (range, 0–109 months). The median time from diagnosis of pulmonary metastases to completion of SBRT was 42 days (range, 21–172 days).

Table 1 summarises clinical data of the patients, lung lesions, and treatment characteristics. All patients had a histopathological confirmation of squamous cell cancer (SCC). The median age at the start of SBRT treatment was 66 years (range, 31–81 years). The majority of patients were male (71.8%). Most patients (76.1%) had an Eastern Co-operative Oncology Group (ECOG) performance score of 0–1. Most of the patients had a history of heavy smoking (67.4% had >20 pack years). The initial tumours were located in the hypopharynx, larynx, nasopharynx, oral cavity, or oral pharynx (8, 11, 2, 4, and 21, respectively). Of the latter, 57.1% had a p16 negative histology. At the initial diagnosis, 28 patients (60.9%) were staged T3–4 according to the TNM classification valid at the respective time. In addition, 28 patients (60.9%) were staged N2–3. Primary treatment of the HNSCC was heterogeneous, with radiotherapy applied in all but 4 patients, either as single modality or as part of a multimodal treatment. Five of the 46 patients (10.9%) had a synchronous metastatic disease, which was treated with SBRT separately from the treatment of the primary. The remaining 41 patients (89.1%) developed their pulmonary lesions later after conclusion of the primary treatment. A total of 17 patients (37.0%) developed new lung metastases after their initial SBRT. These lesions were once more addressed using SBRT, and 2 patients (4.4%) in this subcohort received SBRT even three times for separate lesions. The characteristics of these 17 patients are described in Supplementary Table S1.

A total of 16 patients out of 46 received adjuvant systemic treatment (34.8%) after SBRT. Adjuvant therapy included chemotherapy (CHT) with cetuximab (5 patients, 10.9%), CHT + immune checkpoint inhibitor (ICI) (2 patients, 4.3%), CHT + ICI + cetuximab (4 patients, 8.7%), single-agent CHT (1 patient, 2.2%), cetuximab alone (1 patient, 2.2%), and ICI alone (3 patients, 6.5%). None of the patients received systemic treatment during SBRT.

Of all 92 lesions treated, 48 (52.2%) were present as a single metastasis at the time of SBRT, while the remaining 44 metastases (47.8%) were irradiated in groups of 2 to 4 simultaneously. The most common metastatic sites were the left and the right upper lobes of the lung, accounting for 52.2% of all PMs. The median CTV was 3.4 cm^3^ (range, 0.19–49.55 cm^3^). SBRT dose and fractionation included 45 Gy (3 fractions à 15 Gy; *n =* 61, 66.3%), 48 Gy (6 fractions à 8 Gy; *n* = 15, 16.3%), and 60 Gy (10 fractions à 6 Gy; *n =* 16, 17.4%). The median BED_10_ was 112.5 Gy (range, 86.4–112.5 Gy).

### 3.2. Outcomes

**Figure 1:**
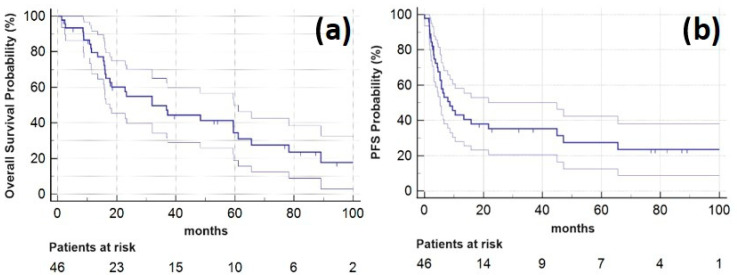
Kaplan–Meier plots showing (**a**) overall survival (horizontal blue line, lighter lines indicating 95% confidence interval) for the study patients following SBRT of the oligometastatic pulmonary lesions and (**b**) progression-free survival for 46 study patients (horizontal blue line, lighter lines indicating 95% confidence interval).

Among all the patients who underwent SBRT, the one-year LC rate, PFS and OS were 98.7% (95% CI: 96.3 to 100.0%), 37.9% (23.7 to 52.1%), and 79.5% (67.6 to 91.5%), respectively, and the two-year LC rate, PFS and OS were 98.7% (96.3 to 100.0%), 28.7% (15.3 to 42.0%), and 54.9% (39.8 to 70.1%). Five years after SBRT LC-rate, PFS and OS were 98.7% (96.3 to 100.0%), 16.7% (5.6 to 27.9%), and 34.5% (18.9 to 50.0%), respectively. Median OS was 32 months (range, 1–109 months; 95% CI: 15.5 to 49.4 months). Kaplan–Meier plots for OS and PFS are provided in Figure 1.

In the univariate analysis, the prognostic factors significantly associated with impaired PFS were radiochemotherapy as primary treatment (vs. surgery followed by radiotherapy; HR = 3.31, *p* = 0.009; Table 2), as well as a reduced patient performance status (ECOG 2 vs. ECOG 0; HR = 2.41, *p* = 0.047). Adjuvant systemic therapy after SBRT is correlated with impaired PFS (HR = 1.77, *p* = 0.096), however, not to a significant extent. An increasing number of metastases treated per patient did not appear to be a potential prognostic factor for PFS, if subcohorts (patient groups with 2, 3, and 4 lesions) are separately analysed and each compared to the reference group (patients with 1 lesion) (see Table 2). By pooling all patients with more than 1 PM in a cumulative subcohort, SBRT of more than 1 lesion turned out to be a significant prognostic factor for PFS (HR = 2.39, *p* = 0.015) in the UVA, but not in the MVA.

In the multivariate analysis, in addition to patient performance status (ECOG 1 vs. ECOG 0, HR = 2.72, *p* = 0.018; ECOG 2 vs. ECOG 0, HR = 2.81, *p* = 0.028; ECOG 3 vs. ECOG 0, HR 48.3, *p* = 0.002) and radiochemotherapy (HR = 5.67, *p* < 0.001), also radiotherapy alone as the primary treatment modality (vs. surgery followed by radiotherapy; HR = 8.6, *p* = 0.003) had a significant impact on PFS. Finally, age (HR = 0.95, *p* = 0.002) was also identified as a significant prognostic factor in the multivariate analysis.

In the univariate analysis of OS, patient performance status (ECOG 3 vs. ECOG 0, HR = 33.19, *p* = 0.005; Table 3) was evidenced to be a significant prognostic factor. Patient performance status (ECOG 3 vs. ECOG 0, HR = 199.51, *p* < 0.001) was also confirmed to have a significant impact on OS in the multivariate analysis. In addition, this analysis identified the primary treatment modality as a second significant prognostic factor for OS (surgery followed by radiochemotherapy vs. surgery followed by radiotherapy, HR = 4.18, *p* = 0.032; radiotherapy vs. surgery followed by radiotherapy, HR = 7.11, *p* = 0.020).

In the univariate analysis, administration of systemic therapy after SBRT appears to decrease OS (HR = 1.57, *p* = 0.230), however, not to a significant extent. The same is valid for an increasing number of metastases treated, if separately analysing subcohorts with different lesion count (ranging from 2 to 4) in comparison to the reference group of patients having only a single SBRT-treated PM (see Table 3). However, analysing the overall survival of a cumulative subcohort (pooled patients with 2–4 PMs), SBRT of more than 1 PM was identified as a prognostic factor for OS in the univariate analysis (HR = 2.76, *p* = 0.017). The finding of more than one metastasis treated by SBRT to be an independent prognostic factor for OS was confirmed in the multivariate analysis (HR = 3.30, *p* = 0.016).

As summarized in Table 2 and Table 3, sex, primary tumour location, UICC stage, smoking history (number of pack years), metastasis timing, p16 status and CTV_total_ did not alter treatment outcome (PFS and OS after SBRT of metastatic lesions).

### 3.3. Treatment-Related Toxicities

The treatment was well tolerated, with only 11 patients (23.9%) developing toxicity of CTCAE grade 1, and 1 patient (2.2%) developing a grade 2 pneumonitis. Spearman’s rank did not show a significant relationship between the mean lung dose (Spearman’s correlation coefficient: 0.201 (−0.012–1.00); *p* = 0.055) and the toxicities observed. Similarly, no significant correlation was found between the cumulative CTV_total_ (1–4 PMs; *p* = 0.359) and the occurrence of adverse effects.

Bone damage was detected in 4 patients (8.7%) by CT imaging during oncological follow-up. These patients did not report on any noticeable symptoms caused by their rib fractures and they did not present at clinics upon pain load. At diagnosis, fractures of all patients were already consolidated.

Of the 46 patients, 5 (11.1%) reported an increase in their pre-existing dyspnoea after SBRT. On the other hand, 2 patients (4.4%) with pre-existing dyspnoea reported an improvement of their respiratory symptoms after SBRT. None of the patients reported on persistent and/or severe fatigue symptoms during follow-up.

For patients who underwent repeated courses of SBRT (*n =* 17, 37.0%) for newly developed pulmonary metastases, spirometry data were available before and after their first SBRT. Of these, 7 patients (41.2%) showed a decrease in the predicted percentage of FEV1 after their first SBRT (mean of −18.7 percentage points). By contrast, 11 patients showed an improvement in their predicted percentage of FEV1 (mean of +8.5 percentage points). Overall, no toxicity higher than grade 2 was observed in our patient cohort. 

## 4. Discussion

Head and neck cancer (HNC) is a highly heterogeneous group of tumours, with squamous cell carcinoma being the predominant histology. Despite significantly improved locoregional control rates and the addition of immunotherapy to the systemic therapy, the median OS of approximately 15 months for patients with metastatic head and neck cancer is still quite poor [34]. During the previous decades, the utilization of local ablation within the context of oligometastatic cancer has gained momentum. Recent studies, such as the SABR-COMET trial [35], have highlighted the potential for enhanced survival in a subgroup of patients with local ablation of metastases. This phase II trial included 99 patients, of whom only 10 had HNSCC with 1–5 metastases. They were randomised to receive palliative standard of care (SOC) or SOC + SBRT to all sites of metastatic disease. The subgroup undergoing local ablation exhibited a significantly improved median OS (41 months vs. 28 months, *p* = 0.09) [35,36] and an 8-year OS rate of 27.2% compared to 13.6% in the SOC control group (*p* = 0.008) [37].

Nevertheless, the evidence for the treatment of pulmonary oligometastatic HNSCC is currently limited to a small number of retrospective studies [22,23,24,25,26,27,28,29], while several randomised trials are still ongoing and lack final reports. Only one randomised trial has examined the role of SBRT in the treatment of patients with exclusively metastatic HNC [38]. However, in this study, Bride et al. focused on the potential abscopal effect of SBRT in combination with Nivolumab on non-irradiated lesions rather than the therapeutic effect of SBRT on irradiated PMs.

With this single-centre, retrospective study, we provide the largest data set to date reporting LC, PFS, and OS of patients with exclusively HNSCC-derived pulmonary metastases (*n =* 92) treated with multiple and partially repeated courses of SBRT. LC rate was excellent with more than 95% after one and still after two years. PFS and OS were approximately 38% and 80% one year and 29% and 55% two years after SBRT. Five years OS was approximately 31%. Median OS reached 32 months after multiple and/or repeated courses of SBRT in comparison to the recently best reported median of 15 months for metastatic HNSCC [34].

Our local control rate (98.7% at two years after SBRT) is in alignment with Pasalic et al. [29], who reported a one-year local control rate of 97.8% and 94.4% two years after SBRT. Even when considering the different tumour type and the much larger sample size of their systematic review, their findings together with ours are in accordance to the reported two-year LC rate of 90% for early stage primary lung cancer treated with SBRT [19]. Bates et al. [25] reported a two-year local control rate of 52% for the subset of metastases situated in the lung. Their study showed a rather poor LC rate in comparison to the higher LC rates reported by Pasalic et al. [29] and the analogously high LC rates presented in our study. One reason for this significant inconsistency could be explained by the fact that Bates et al. treated 91.7% of all metastases with a BED10 of less than 100 Gy, whereas 66.3% of the metastases in our study were irradiated with a BED10 of 112.5 Gy. An identical BED10 was used by Pasalic et al. in the treatment of 73.8% of all their PMs. For SBRT of non-small-cell lung cancer it is known that a BED10 ≥ 100 Gy is associated with significantly better local control (and survival) than less intensive radiation regimes [39,40]. Recently, Said et al. confirmed the findings of Ricco et al. [41] in their retrospective study by clearly demonstrating the beneficial effect of a BED10 ≥ 100 Gy on the local control rate of HNC-derived PMs treated with SBRT [28].

The two-year OS rate of 54.9% for metastatic HNSCC assessed in our patient cohort is in agreement with Pasalic et al. [29], who reported a two-year OS of 55% for the subgroup of patients (HNSCC-derived pulmonary lesions only) treated with SBRT. Our results are also comparable, and even higher than the reported respective OS for patients with metastatic HNSCC after pulmonary metastasectomy (~50% determined by Shiono et al. [42]). Considering the invasiveness of metastasectomy—Shiono et al. reported a lobectomy rate of 54% [42]—and the perioperative risk, associated rehabilitation efforts as well as treatment and hospitalisation costs, SBRT should be further evaluated in larger trials as a beneficial treatment option for all patients with HNSCC-derived pulmonary metastases, viz., for inoperable and resectable PMs. In addition, SBRT offers the opportunity to treat lesions at multiple sites simultaneously. However, there are no ongoing randomised trials systematically investigating benefits and shortages of SBRT versus metastasectomy in HNSCC patients. In summary, OS in previously published retrospective studies using either surgery or SBRT reaches up to 75% at 1 year and 40–50% at 5 years after treatment [36].

SBRT for HNC-derived PMs is a safe treatment option with reported toxicities not higher than grade 2 [24,26,27,29], which was confirmed by the data analysed in the present cohort. This finding was further supported by available spirometry data from our department, demonstrating that no patient treated with SBRT suffered from functional impairment of the lung. Rib fractures occurred in 4 patients of our cohort (8.7%); however, all were consolidated within the follow-up period. Rib fractures are a common toxicity following SBRT of lung tumours or pulmonary metastases with dose-dependent incidences up to 40% [43,44].

Even repeated courses of SBRT for multiple pulmonary lesions was demonstrated to be safe in a subcohort of 145 patients with metastases of different origin [45,46]. Also, re-SBRT of local recurrences of NSCLC situated within the 25% isodose of a previously performed SBRT did not lead to toxicities ≥grade 3, as it has been observed by Kennedy et al. [47]. In general, it should be mentioned that direct comparison of outcomes, LC, and treatment-associated toxicities between the various published studies on SBRT of oligometastatic disease is hampered by the often-heterogeneous composition of patients with different primary tumours and different systemic therapies.

To our knowledge, our study is the first to report on outcome and toxicity in a subgroup of patients who received multiple and repeated courses of SBRT treatments for HNSCC-derived pulmonary metastases only. With this approach, excellent local control was achieved without increased incidence and severity of toxicities even after repeated courses of SBRT.

In the investigated cohort, we identified patient performance status and the primary treatment modality as significant predictors of PFS and OS. Other retrospective studies identified the CTV/PTV size [27,28], the patient performance status [27], spinal disease [27], and the use of adjuvant systemic therapy [29] as prognostic factors for PFS and/or OS. Also in our cohort, the administration of systemic therapy after SBRT (34.8% of all patients) is accompanied by a considerable reduction in PFS and OS, however, not to a statistically significant extent. Under no circumstances should the observed reduction in PFS and OS be interpreted to discourage the use of adjuvant systemic therapy, since it is primarily utilized in patients with progressive systemic disease and with a, therefore, inherently impaired outcome prognosis.

When analysing the overall survival of patients treated with SBRT for 2 to 4 pulmonary metastases, it was observed that treating more than 1 metastasis emerged as a prognostic factor for OS. The analysis indicates that when multiple metastatic lesions are treated with SBRT, this is linked to a threefold higher risk of reduced overall survival if compared to patients treated for a single PM. This association is statistically significant in both univariate and multivariate analyses, highlighting multiple pulmonary metastases (≥2) as an independent predictor of impaired overall survival.

In other studies, additional factors, such as patient age and sex, absence or presence of brain or bone metastases, oral cavity or sinonasal location of the primary, molecular markers, and genetic alterations such as HPV status, are either recognised or still being discussed as potential outcome predictors [28].

The identification and systematic removal of confounding factors might contribute to the assessment of which of the prognostic parameters under discussion, finally, will meet the required level of robustness. This topic should also be addressed in randomised trials, which, in future, might further outline the potential risks and benefits associated with multiple and/or repeated courses of SBRTs of PMs, in order to reliably select patients who will really profit most from this advanced treatment option.

Given its retrospective nature, encountered limitations of the present study include unavoidable selection bias and the possibility of incomplete documentation. Furthermore, our retrospective analysis was limited by incomplete assessment of primary molecular markers and HPV data. Although we present the largest data set on exclusively HNSCC-derived PMs so far, which were analysed over a period of 15 years, the sample size in our single-centre study is still limited (*n =* 92 PMs). In particular, our (near-significant) results regarding the analysis of adjuvant systemic treatment, as well as those of the separate analysis of subcohorts with different counts of treated metastases, could both benefit from larger sample sizes, in order to clearly define the potential influence of these factors on PFS and OS. This also applies to our results regarding the correlation between mean lung dose and observed toxicities.

Within the retrospective period in this study, remarkable progress has been achieved in imaging, systemic treatment, and SBRT techniques, which limits a homogeneous comparison of patients treated over time. As previously mentioned by other authors [25,28,29], uncertainties potentially arise also in clearly identifying separate histological origins (HNSCC vs. squamous cell carcinoma of the lung) of the treated squamous lesions due to the lacking data of unambiguous molecular identification.

## 5. Conclusions

Taken together, the demonstrated feasibility of SBRT and its favourable local control rate and overall survival provide evidence to recommend SBRT as a safe and beneficial treatment option for patients with pulmonary oligometastatic HNSCC. Multiple and repeated courses of SBRT for newly emerged pulmonary metastases is effective and well tolerated. Future randomised trials enrolling more patients with oligometastatic HNSCC are needed to identify independent prognostic factors and to investigate the potential and safety of SBRT in combination with systemic treatments.

## Figures and Tables

**Table 1:** Patient, lesion, and treatment characteristics.

Characteristic		Value or No. (%)
Total no. of patients		46
Total no. of metastases		92
Median age at start or SBRT (range)		66 (31–81)
Sex	Male Female	33 (71.8%) 13 (28.2%)
Performance status	0 1 2 3	20 (43.5%) 15 (32.6%) 10 (21.7%) 1 (2.2%)
Pack years	Not available 0 <10 10–20 >20	1 (2.2%) 4 (8.7%) 4 (8.7%) 6 (13%) 31 (67.4%)
Primary tumour location	Hypopharynx Larynx Nasopharynx Oral cavity Oropharynx	8 (17.4%) 11 (23.9%) 2 (4.4%) 4 (8.7%) 21 (45.6%)
p16 status (oropharyngeal cancer only)	Not available Positive Negative	6 (28.6%) 3 (14.3%) 12 (57.1%)
AJCC tumour classification (valid at the time of diagnosis)	T1 T2 T3 T4	5 (10.8%) 13 (28.3%) 12 (26.1%) 16 (34.8%)
AJCC nodal classification (valid at the time of diagnosis)	N0 N1 N2 N3	10 (21.7%) 8 (17.4%) 26 (56.5%) 2 (4.4%)
Primary treatment	Surgery Surgery + RT Surgery + RCHT RT RCHT	4 (8.7%) 12 (26.1%) 12 (26.1%) 4 (8.7%) 14 (30.4%)
Metastasis timing	Metachronous Synchronous	41 (89.1%) 5 (10.9%)
Number of different SBRT	1 2 3	29 (63.0%) 15 (32.6%) 2 (4.4%)
Treated lesions	1 Lesions per SBRT 2 Lesions per SBRT 3 Lesions per SBRT 4 Lesions per SBRT	92 48 (52.2%) 10 (=20) (21.8%) 4 (=12) (13.0%) 3 (=12) (13.0%)
Laterality	Left lower lobe Left upper lobe Right lower lobe Right middle lobe Right upper lobe	14 (15.2%) 25 (27.2%) 21 (22.8%) 9 (9.8%) 23 (25%)
Median clinical tumour volume, cm³ (range)		3.40 (0.19; 49.55)
Dose, Gy/fraction number	60/10 48/6 45/3	16 (17.4%) 15 (16.3%) 61 (66.3%)
Adjuvant systemic therapy in total of which: CHT + Cetuximab CHT + ICI CHT + Cetuximab + ICI Single-agent CHT Cetuximab alone ICI alone		16 (34.8%) 5 (10.9%) 2 (4.3%) 4 (8.7%) 1 (2.2%) 1 (2.2%) 3 (6.5%)

AJCC: American Joint Committee on Cancer; RCHT: radiochemotherapy; RT: radiotherapy; ICI: immune checkpoint inhibitor; CHT: chemotherapy (various combinations of Cisplatin, Carboplatin, 5-FU, Docetaxel, and Methotrexat).

**Table 2:** Univariate and multivariate Analysis of factors related to PFS. Significant *p* values are in bolds.

Univariate Hazard Ratio for PFS			Multivariate Hazard Ratio for PFS		
Factor	HR (CI 95%)	*p* Value		HR (CI 95%)	*p* Value
Sex Male Female	1 (reference) 1.27 (0.66–2.48)	0.474			
Age	0.98 (0.94–1.07)	0.116		0.95 (0.91–0.98)	**0.002**
Primary tumour location Oropharynx Hypopharynx Larynx Nasopharynx Oral cavity	1 (reference) 0.80 (0.13–4.90) 1.72 (0.66–4.49) 1.67 (0.37–7.36) 1.05 (0.30–3.67)	0.900 0.176 0.487 0.934			
Stage UICC I II III IV	1 (reference) 0.80 (0.13–4.90) 0.63 (0.17–2.27) 1.14 (0.34–3.84)	0.807 0.477 0.837			
Primary treatment Surgery + RT Surgery Surgery + RCHT RCHT RT	1 (reference) 1.61 (0.48–5.36) 1.72 (0.66–4.49) 3.31 (1.35–8.11) 2.64 (0.79–8.84)	0.441 0.265 **0.009** 0.116		1 (reference) 3.10 (0.85–11.34) 2.17 (0.76–6.25) 5.67 (2.03–15.84) 8.60 (2.11–35.04)	0.087 0.150 **<0.001** **0.003**
Pack years 0 <10 10–20 >20	1 (reference) 0.96 (0.24–3.91) 1.18 (0.33–4.25) 0.60 (0.21–1.76)	0.959 0.796 0.349			
Performance status (ECOG) 0 1 2 3	1 (reference) 1.50 (0.72–3.11) 2.41 (1.01–5.73) 5.72 (0.69–47.1)	0.280 **0.047** 0.105		1 (reference) 2.72 (1.19–6.21) 2.81 (1.12–7.05) 48.30 (4.13–565.02)	**0.018** **0.028** **0.002**
Metastasis timing Metachronous Synchronous	1 (reference) 1.29 (0.45–3.71)	0.642			
p16 status Negative Positive	1 (reference) 1.14 (0.30–4.25)	0.850			
Adjuvant systemic therapy No yes	1 (reference) 1.77 (0.90–3.46)	0.096			
CTV_total_	1.007 (0.982–1.033)	0.590			
Number of treated metastases 1 2 3 4	1 (reference) 2.38 (0.99–5.28) 1.91 (0.56–6.50) 4.15 (0.92–18.71)	0.053 0.302 0.064			
Number of treated metastases 1 >1 (range 2–4)	1 (reference) 2.39 (1.19–4.79)	**0.015**			

**Table 3:** Univariate and multivariate analysis of factors related to OS. Significant *p* values are in bolds.

Univariate Hazard Ratio for OS			Multivariate Hazard Ratio for OS		
Factor	HR (CI 95%)	*p* Value		HR (CI 95%)	*p* Value
Sex Male Female	1 (reference) 0.58 (0.27–1.21)	0.146			
Age	1.01 (0.97–1.04)	0.761			
Primary tumour location Oropharynx Hypopharynx Larynx Oral cavity	1 (reference) 2.04 (0.75–5.55) 1.06 (0.45–2.47) 0.86 (0.19–3.88)	0.161 0.901 0.845			
Stage UICC I II III IV	1 (reference) 1.18 (0.16–8.56) 0.413 (0.08–2.04) 0.877 (0.20–3.83)	0.869 0.277 0.862			
Primary treatment Surgery + RT Surgery Surgery + RCHT RCHT RT	1 (reference) 1.90 (0.46–7.85) 2.46 (0.83–7.28) 1.91 (0.68–5.33) 3.04 (0.74–12.4)	0.374 0.105 0.218 0.122		1 (reference) 4.55 (0.94–22.10) 4.18 (1.13–15.46) 2.26 (0.67–7.69) 7.11 (1.37–37.09)	0.060 **0.032** 0.191 **0.020**
Pack years 0 <10 10–20 >20	1 (reference) 1.68 (0.37–7.62) 1.04 (0.24–4.48) 0.60 (0.17–2.10)	0.958 0.958 0.423			
Performance status (ECOG) 0 1 2 3	1 (reference) 1.67 (0.70–3.98) 2.57 (0.98–6.71) 33.19 (2.83–388.66)	0.245 0.054 **0.005**		1 (reference) 2.30 (0.82–6.46) 2.74 (0.94–7.98) 195.51 (11.86–3324.03)	0.115 0.065 **<0.001**
Metastasis timing Metachronous Synchronous	1 (reference) 1.29 (0.45–3.71)	0.642			
p16 status Negative Positive	1 (reference) 0.588 (0.07–5.11)	0.630			
Adjuvant systemic therapy No Yes	1 (reference) 1.57 (0.75–3.28)	0.230			
CTV_total_	1.012 (0.986–1.039)	0.373			
Number of treated metastases 1 2 3 4	1 (reference) 2.47 (0.96–6.37) 3.40 (0.95–12.12) 3.84 (0.46–32.40)	0.061 0.059 0.216			
Number of treated metastases 1 >1 (range 2–4)	1 (reference) 2.76 (1.19–6.36)	**0.017**		1 (reference) 3.30 (1.25–8.68)	**0.016**

## Data Availability

The data presented in this study are available on request from the corresponding author.

## Supplementary Materials

The following supporting information can be downloaded at: https://www.mdpi.com/article/10.3390/cancers15215253/s1, Table S1: Patient, lesion, and treatment characteristics of repeated courses of SBRT.

## References

[1] Kawakita D., Oze I., Iwasaki S., Matsuda T., Matsuo K., Ito H. (2022). Trends in the Incidence of Head and Neck Cancer by Subsite between 1993 and 2015 in Japan. Cancer Med..

[2] Sung H., Ferlay J., Siegel R.L., Laversanne M., Soerjomataram I., Jemal A., Bray F. (2021). Global Cancer Statistics 2020: GLOBOCAN Estimates of Incidence and Mortality Worldwide for 36 Cancers in 185 Countries. CA Cancer J. Clin..

[3] Hermanns I., Ziadat R., Schlattmann P., Guntinas-Lichius O. (2021). Trends in Treatment of Head and Neck Cancer in Germany: A Diagnosis-Related-Groups-Based Nationwide Analysis, 2005–2018. Cancers.

[4] Forastiere A.A., Ismaila N., Lewin J.S., Nathan C.A., Adelstein D.J., Eisbruch A., Fass G., Fisher S.G., Laurie S.A., Le Q.T. (2018). Use of Larynx-Preservation Strategies in the Treatment of Laryngeal Cancer: American Society of Clinical Oncology Clinical Practice Guideline Update. J. Clin. Oncol..

[5] Pignon J.P., Le Maitre A., Maillard E., Bourhis J., Group M.-N.C. (2009). Meta-Analysis of Chemotherapy in Head and Neck Cancer (MACH-NC): An Update on 93 Randomised Trials and 17,346 Patients. Radiother. Oncol..

[6] Hakansson K., Rasmussen J.H., Bentzen S.M., Friborg J., Specht L., Vogelius I.R. (2019). On the Relation between Improved Loco-regional Control and Disease-Free Survival in Head-and-Neck Cancer. Acta Oncol..

[7] Teoh M., Clark C.H., Wood K., Whitaker S., Nisbet A. (2011). Volumetric Modulated Arc Therapy: A Review of Current Literature and Clinical Use in Practice. Br. J. Radiol..

[8] Spence T., Bruce J., Yip K.W., Liu F.F. (2016). HPV Associated Head and Neck Cancer. Cancers.

[9] Fakhry C., Gillison M.L. (2006). Clinical Implications of Human Papillomavirus in Head and Neck Cancers. J. Clin. Oncol..

[10] Liu J.C., Bhayani M., Kuchta K., Galloway T., Fundakowski C. (2019). Patterns of Distant Metastasis in Head and Neck Cancer at Presentation: Implications for Initial Evaluation. Oral Oncol..

[11] Vermorken J.B., Specenier P. (2010). Optimal Treatment for Recurrent/Metastatic Head and Neck Cancer. Ann. Oncol..

[12] Duprez F., Berwouts D., De Neve W., Bonte K., Boterberg T., Deron P., Huvenne W., Rottey S., Mareel M. (2017). Distant Metastases in Head and Neck Cancer. Head Neck.

[13] Haigentz M., Hartl D.M., Silver C.E., Langendijk J.A., Strojan P., Paleri V., de Bree R., Machiels J.P., Hamoir M., Rinaldo A. (2012). Distant Metastases from Head and Neck Squamous Cell Carcinoma. Part III. Treatment. Oral Oncol..

[14] Sacco A.G., Cohen E.E. (2015). Current Treatment Options for Recurrent or Metastatic Head and Neck Squamous Cell Carcinoma. J. Clin. Oncol..

[15] D’Onofrio I., Nardone V., Reginelli A., Cappabianca S. (2023). Chemoradiotherapy for Head and Neck Cancer. Cancers.

[16] Hellman S., Weichselbaum R.R. (1995). Oligometastases. J. Clin. Oncol..

[17] Lievens Y., Guckenberger M., Gomez D., Hoyer M., Iyengar P., Kindts I., Mendez Romero A., Nevens D., Palma D., Park C. (2020). Defining Oligometastatic Disease from a Radiation Oncology Perspective: An ESTRO-ASTRO Consensus Document. Radiother. Oncol..

[18] Lo S.S., Fakiris A.J., Chang E.L., Mayr N.A., Wang J.Z., Papiez L., Teh B.S., McGarry R.C., Cardenes H.R., Timmerman R.D. (2010). Stereotactic Body Radiation Therapy: A Novel Treatment Modality. Nat. Rev. Clin. Oncol..

[19] Murray P., Franks K., Hanna G.G. (2017). A Systematic Review of Outcomes Following Stereotactic Ablative Radiotherapy in the Treatment of Early-Stage Primary Lung Cancer. Br. J. Radiol..

[20] Ferlito A., Shaha A.R., Silver C.E., Rinaldo A., Mondin V. (2001). Incidence and Sites of Distant Metastases from Head and Neck Cancer. ORL J. Otorhinolaryngol. Relat. Spec..

[21] Kotwall C., Sako K., Razack M.S., Rao U., Bakamjian V., Shedd D.P. (1987). Metastatic Patterns in Squamous Cell Cancer of the Head and Neck. Am. J. Surg..

[22] Vincent A.G., Wang W., Shokri T., Ducic Y. (2021). Treatment of Oligometastatic Disease in Squamous Cell Carcinoma of the Head and Neck. Laryngoscope.

[23] Weissmann T., Hofler D., Hecht M., Semrau S., Haderlein M., Filimonova I., Frey B., Bert C., Lettmaier S., Mantsopoulos K. (2021). Oligometastatic Head and Neck Cancer: Which Patients Benefit from Radical Local Treatment of All Tumour Sites?. Radiat. Oncol..

[24] Franzese C., Badalamenti M., Teriaca A., De Virgilio A., Mercante G., Cavina R., Ferrari D., Santoro A., Spriano G., Scorsetti M. (2021). Metastasis-Directed Stereotactic Body Radiation Therapy in the Management of Oligometastatic Head and Neck Cancer. J. Cancer Res. Clin. Oncol..

[25] Bates J.E., De Leo A.N., Morris C.G., Amdur R.J., Dagan R. (2019). Oligometastatic Squamous Cell Carcinoma of the Head and Neck Treated with Stereotactic Body Ablative Radiotherapy: Single-Institution Outcomes. Head Neck.

[26] Bonomo P., Greto D., Desideri I., Loi M., Di Cataldo V., Orlandi E., Iacovelli N.A., Becherini C., Visani L., Salvestrini V. (2019). Clinical Outcome of Stereotactic Body Radiotherapy for Lung-Only Oligometastatic Head and Neck Squamous Cell Carcinoma: Is the Deferral of Systemic Therapy a Potential Goal?. Oral Oncol..

[27] Singh R., Jenkins J., Davis J., Song S., Sharma S., Vargo J.A. (2022). A Multi-Institutional Analysis of Outcomes Following Stereotactic Body Radiation Therapy for Management of Metastases from Squamous Cell Carcinomas of the Head and Neck. J. Radiosurg. SBRT.

[28] Id Said B., Mutsaers A., Chen H., Husain Z.A., Biswas T., Dagan R., Erler D., Foote M., Louie A.V., Redmond K. (2023). Outcomes for Oligometastatic Head and Neck Cancer Treated with Stereotactic Body Radiotherapy: Results from an International Multi-Institutional Consortium. Head Neck.

[29] Pasalic D., Betancourt-Cuellar S.L., Taku N., Ludmir E.B., Lu Y., Allen P.K., Tang C., Antonoff M.B., Fuller C.D., Rosenthal D.I. (2020). Outcomes and Toxicities Following Stereotactic Ablative Radiotherapy for Pulmonary Metastases in Patients with Primary Head and Neck Cancer. Head Neck.

[30] Azam F., Latif M.F., Farooq A., Tirmazy S.H., AlShahrani S., Bashir S., Bukhari N. (2019). Performance Status Assessment by Using ECOG (Eastern Cooperative Oncology Group) Score for Cancer Patients by Oncology Healthcare Professionals. Case Rep. Oncol..

[31] Dejaco D., Steinbichler T., Schartinger V.H., Fischer N., Anegg M., Dudas J., Posch A., Widmann G., Riechelmann H. (2019). Specific Growth Rates Calculated from CTS in Patients with Head and Neck Squamous Cell Carcinoma: A Retrospective Study Performed in Austria. BMJ Open.

[32] Eisenhauer E.A., Therasse P., Bogaerts J., Schwartz L.H., Sargent D., Ford R., Dancey J., Arbuck S., Gwyther S., Mooney M. (2009). New Response Evaluation Criteria in Solid Tumours: Revised RECIST Guideline (version 1.1). Eur. J. Cancer.

[33] Trotti A., Colevas A.D., Setser A., Rusch V., Jaques D., Budach V., Langer C., Murphy B., Cumberlin R., Coleman C.N. (2003). CTCAE v3.0: Development of a Comprehensive Grading System for the Adverse Effects of Cancer Treatment. Semin. Radiat. Oncol..

[34] Burtness B., Harrington K.J., Greil R., Soulieres D., Tahara M., de Castro G., Psyrri A., Baste N., Neupane P., Bratland A. (2019). Pembrolizumab Alone or with Chemotherapy Versus Cetuximab with Chemotherapy for Recurrent or Metastatic Squamous Cell Carcinoma of the Head and Neck (KEYNOTE-048): A Randomised, Open-Label, Phase 3 Study. Lancet.

[35] Palma D.A., Olson R., Harrow S., Gaede S., Louie A.V., Haasbeek C., Mulroy L., Lock M., Rodrigues G.B., Yaremko B.P. (2019). Stereotactic Ablative Radiotherapy Versus Standard of Care Palliative Treatment in Patients with Oligometastatic Cancers (SABR-COMET): A Randomised, Phase 2, Open-Label Trial. Lancet.

[36] Bahig H., Huang S.H., O’Sullivan B. (2022). Oligometastatic Head and Neck Cancer: Challenges and Perspectives. Cancers.

[37] Harrow S., Palma D.A., Olson R., Gaede S., Louie A.V., Haasbeek C., Mulroy L., Lock M., Rodrigues G.B., Yaremko B.P. (2022). Stereotactic Radiation for the Comprehensive Treatment of Oligometastases (SABR-COMET): Extended Long-Term Outcomes. Int. J. Radiat. Oncol. Biol. Phys..

[38] McBride S., Sherman E., Tsai C.J., Baxi S., Aghalar J., Eng J., Zhi W.I., McFarland D., Michel L.S., Young R. (2021). Randomized Phase II Trial of Nivolumab With Stereotactic Body Radiotherapy Versus Nivolumab Alone in Metastatic Head and Neck Squamous Cell Carcinoma. J. Clin. Oncol..

[39] Onishi H., Shirato H., Nagata Y., Hiraoka M., Fujino M., Gomi K., Niibe Y., Karasawa K., Hayakawa K., Takai Y. (2007). Hypofractionated Stereotactic Radiotherapy (HypoFXSRT) for Stage I Non-Small Cell Lung Cancer: Updated Results of 257 Patients in a Japanese Multi-Institutional Study. J. Thorac. Oncol..

[40] Moreno A.C., Fellman B., Hobbs B.P., Liao Z., Gomez D.R., Chen A., Hahn S.M., Chang J.Y., Lin S.H. (2020). Biologically Effective Dose in Stereotactic Body Radiotherapy and Survival for Patients with Early-Stage NSCLC. J. Thorac. Oncol..

[41] Ricco A., Davis J., Rate W., Yang J., Perry D., Pablo J., D’Ambrosio D., Sharma S., Sundararaman S., Kolker J. (2017). Lung Metastases Treated with Stereotactic Body Radiotherapy: The RSSearch^®^ Patient Registry’s Experience. Radiat. Oncol..

[42] Shiono S., Kawamura M., Sato T., Okumura S., Nakajima J., Yoshino I., Ikeda N., Horio H., Akiyama H., Kobayashi K. (2009). Pulmonary Metastasectomy for Pulmonary Metastases of Head and Neck Squamous cell Carcinomas. Ann. Thorac. Surg..

[43] Kim S.S., Song S.Y., Kwak J., Ahn S.D., Kim J.H., Lee J.S., Kim W.S., Kim S.W., Choi E.K. (2013). Clinical Prognostic Factors and Grading System for Rib Fracture Following Stereotactic Body Radiation Therapy (SBRT) in Patients with Peripheral Lung Tumors. Lung Cancer.

[44] Carducci M.P.S., Sundaram B., Greenberger B.A., Werner-Wasik M., Kane G.C. (2023). Predictors and Characteristics of Rib Fracture Following SBRT for Lung Tumors. BMC Cancer.

[45] Klement R.J., Hoerner-Rieber J., Adebahr S., Andratschke N., Blanck O., Boda-Heggemann J., Duma M., Eble M.J., Eich H.C., Flentje M. (2018). Stereotactic Body Radiotherapy (SBRT) for Multiple Pulmonary Oligometastases: Analysis of Number and Timing of Repeat SBRT as Impact Factors on Treatment Safety and Efficacy. Radiother. Oncol..

[46] Owen D., Olivier K.R., Mayo C.S., Miller R.C., Nelson K., Bauer H., Brown P.D., Park S.S., Ma D.J., Garces Y.I. (2015). Outcomes of Stereotactic Body Radiotherapy (SBRT) Treatment of Multiple Synchronous and Recurrent Lung Nodules. Radiat. Oncol..

[47] Kennedy W.R., Gabani P., Nikitas J., Robinson C.G., Bradley J.D., Roach M.C. (2020). Repeat Stereotactic Body Radiation Therapy (SBRT) for Salvage of Isolated Local Recurrence after Definitive Lung SBRT. Radiother. Oncol..

