# Supplementary material for: Survival, Treatment Outcome, and Safety of Multiple and Repeated Courses of Stereotactic Body Radiotherapy for Pulmonary Oligometastases of Head and Neck Squamous Cell Carcinoma
Source: Cancers (Basel). 2023 Nov 1;15(21):5253. doi: 10.3390/cancers15215253 (PMC10647772; doi:10.3390/cancers15215253)
Supplement: Supplementary file 1 [file cancers-15-05253-s001.zip › cancers-2653606-supplementary.pdf]

Table S1. Patient, lesions, and treatment characteristics of repeated courses of SBRT.

Characteristic		Value or Ndo. (%)
**Total no. of patients**		17
**Total no. of metastases**		46
**Median age at start or SBRT (range)**		68 (44-79)
**Sex**	Male	15 (88.2%)
	Female	2 (11.8%)
**Performance Status**	0	8 (47.1%)
	1	4 (23.5%)
	2	5 (29.4%)
**Pack years**	Not available	1 (5.9%)
	0	1 (5.9%)
	< 10	0
	10-20	4 (23.5%)
	> 20	11 (64.7%)
**Primary tumour location**	Hypopharynx	2 (11.8%)
	Larynx	4 (23.5%)
	Nasopharynx	2 (11.8%)
	Oral cavity	2 (11.8%)
	Oropharynx	7 (41.2%)
**AJCC tumour classification (valid at the time of diagnosis)**	T1	1 (5.9%)
	T2	6 (35.3%)
	T3	5 (29.4%)
	T4	5 (29.4%)
**AJCC nodal classification (valid at the time of diagnosis)**	N0	3 (17.6%)
	N1	3 (17.6%)
	N2	10 (58.8%)
	N3	1 (5.9%)
**Primary treatment**	Surgery	1 (5.9%)
	Surgery + RT	5 (29.4%)
	Surgery + RCHT	4 (23.5%)
	RCHT	7 (41.2%)
**Metastasis timing**	Metachronous	15 (88.2%)
	Synchronous	2 (11.8%)
**Number of different SBRT**	2	15 (88.2%)
	3	2 (11.8%)
**Treated Lesions**		46
	1 Lesions per SBRT	29 (63.1%)
	2 Lesions per SBRT	5 (=10) (21.7%)
	3 Lesions per SBRT	1 (=3) 6.5%)
	4 Lesions per SBRT	1 (=4) (8.7%)
**Laterality**	Left lower lobe	16 (15.5%)
	Left upper lobe	23 (22.3%)
	Right lower lobe	23 (22.3%)
	Right middle lobe	10 (9.7%)
	Right upper lobe	31 (30.1%)
**Median clinical tumour volume, cm³ (range)**		3,21 (0.17; 49.62)

**Dose, Gy/fraction number**	60/10	14 (13.6%)
	48/6	20 (19.4%)
	45/3	69 (67%)
**Adjuvant systemic therapy in total**		6 (35.3%)
**of which:**		
CHT + ICI		2 (11.8%)
CHT + Cetuximab + ICI		1 (5.9%)
ICI alone		3 (17.6%)

RCHT: radiochemotherapy; RT: radiotherapy; ICI: immune checkpoint inhibitor; CHT: chemotherapy (various combinations of Cisplatin, Carboplatin, 5-FU, Docetaxel, and Methotrexat).
